# Proteomics and genomics of a monomorphic epitheliotropic intestinal T-cell lymphoma: An extremely rare case report and short review of literature

**DOI:** 10.1097/MD.0000000000031951

**Published:** 2022-11-25

**Authors:** Mădălina Boșoteanu, Miruna Cristian, Mariana Așchie, Mariana Deacu, Anca Florentina Mitroi, Costel Stelian Brînzan, Gabriela Izabela Bălțătescu

**Affiliations:** a Faculty of Medicine, “Ovidius” University of Constanta, Romania; b Department of Clinical Pathology, “Sf. Apostol Andrei” Emergency County Hospital, Constanta, Romania; c Center for Research and Development of the Morphological and Genetic Studies of Malignant Pathology - CEDMOG, “Ovidius” University of Constanta, Romania; d Academy of Medical Sciences, Bucharest, Romania.

**Keywords:** case report, lymphoma, non-Hodgkin, proto-oncogene protein pim-1

## Abstract

Monomorphic epitheliotropic intestinal T-cell lymphoma, formerly known as enteropathy-associated T-cell lymphoma, is an extremely rare, aggressive peripheral extranodal T-cell lymphoma, that is infrequent in native European and Caucasian populations. The current study presents the clinicopathological features, diagnostic approach, and clinical outcomes of this rare entity of lymphoma and highlights the importance of the early diagnosis of monomorphic epitheliotropic intestinal T-cell lymphoma (MEITL).

**Patient concerns:**

Main symptoms and/or important clinical findings: We present the case of a 69-year-old male patient presenting with an abdominal mass, intestinal transit disorder, and weight loss. The abdominal computed tomography (CT) revealed features suggestive of a malignancy. Following clinical and imaging investigations, surgical resection of the small intestine with other areas of involvement has been performed and further to the histopathological examination and immunohistochemical testing are mandatory.

**Diagnoses and Interventions:**

Histopathological evaluation of the tumor revealed a proliferation of medium- to large-sized monomorphic lymphocytes, with vesicular nuclei, prominent nucleoli, and a moderate amount of clear to pale eosinophilic cytoplasm, with an association of infrequent Reed-Sternberg-like cells. Immunohistochemical assessment of the aforementioned tumor using CD3, CD8, CD5, CD20, and CD30 confirmed the T cell proliferation line and the monomorphic epitheliotropic intestinal T-cell lymphoma diagnosis.

**Lessons:**

The current report highlights the importance of early diagnosis of MEITL owing to its poor prognosis and presents histopathological features that help distinguish MEITL from inflammatory bowel diseases and less aggressive T-cell lymphomas.

## 1. Introduction

Monomorphic epitheliotropic intestinal T-cell lymphoma (MEITL) (formerly termed enteropathy-associated T cell lymphoma [EATL], type II) is an extremely rare peripheral T-cell lymphoma in South-Eastern Romania, derived from intraepithelial T cells in the gastrointestinal (GI) tract and unlike the classic form of EATL, tends to behave aggressively and there is no clear association with celiac disease and/or other malabsorption syndromes and inflammatory colitis.^[1,2]^ MEITL was previously subclassified as EATL type 2 and based on distinctive pathological and epidemiological features, and to help distinction from EATL, this disease is no longer referred to as type 1 EATL.^[1]^ Most often MEITL involves the small bowel, particularly the jejunum and ileum, but it can also involve the stomach, colon, and other extraintestinal sites.^[3]^ Histologically, the tumor typically consists of small- to medium-sized monomorphic lymphocytes with hyperchromatic nuclei with inconspicuous nucleoli and a moderate amount of clear to pale eosinophilic cytoplasm and the mitotic activity is mostly brisk, and unlike EATL, there usually is no significant inflammatory background or necrosis.^[4,5]^

Its clinical, morphologic, and immunophenotypic features distinguishing it from the more common EATL (previously EATL type I) make it a separate entity and due to its poor prognosis, it needs to be distinguished from inflammatory diseases and less aggressive T-cell lymphoma.^[2,6]^ MEITL has a worldwide distribution, and it accounts for the vast majority of cases of primary intestinal T-cell lymphoma occurring in Asia and which also appears to occur with increased frequency in males, who are affected more often than females, the male-to-female ratio being approximately 2:1.^[1]^ The small intestine is the most often involved in this type of lymphoma, with the jejunum affected more often than the ileum.

The clinical outcome of patients with MEITL is poor, with a median survival of 7 months and 5 years overall and complete response rates are poor: 46% and 48%, respectively.^[1]^

According to a study of Delabie J. et al (2011), elevated serum lactate dehydrogenase (LDH) and C-reactive protein (CRP) levels are risk factors associated with worse overall survival and failure-free survival in MEITL/EATL patients.^[3]^

Neoplastic cells typically display the CD8^ + ^cytotoxic phenotype and are often CD30^−^; whereas Type I EATL is usually CD8^−^ and at least focally positive for CD30.^[4]^

The Proviral Integration site of the Moloney murine leukemia virus (PIM) family is an important mediator of cell survival, comprising 3 ubiquitously expressed serine/threonine kinases (PIM1, PIM2, and PIM3) with a broad range of cellular substrates that promote cell growth, proliferation, and drug resistance and, they are overexpressed in a number of human cancers and frequently associated with poor prognosis in most hematological malignancies.^[7,8]^ The identification as cooperating targets of Proviral Integration of Moloney virus in murine lymphomas suggested early on that PIM serine/threonine kinases play a significant role in cancer biology.^[9]^

Typically, PIM kinases are activated by signaling pathways downstream of growth factors, cytokines, and mitogenic stimuli, such as the Janus Kinase-signal transducer and activator of transcription and nuclear factor Kappa B, specimen proof 70 and heat shock protein 90 also shield them from proteasomal breakdown.^[7,9,10]^ They function by phosphorylating a wide range of proteins, including those that control transcription (MYC, MYB, RUNX1, RUNX3), cell cycle (p21, p27, CDC25A, CDC25C), protein translation (eukaryotic translation initiation factor 4E-binding protein 1), apoptosis, signaling intermediates (SOCS1, SOCS3, MAP3K5, mTOR, AKT), and drug resistance proteins.

PIM kinases collaborate with crucial genes involved in B- and T-cell lymphomagenesis, including c-MYC, B-cell lymphoma 6, and E2A-PBX1, according to studies using transgenic mice.^[9]^

The main purpose of the current study is to report a case of a Caucasian male patient who presented with chronic GI symptoms and normal levels of LDH and CRP at diagnosis, who was found to have MEITL upon resection of the small intestine with multiple areas of involvement noted by imagistic investigations. Moreover, we aimed to find the presence or absence of PIM1 kinase with real time polymerase chain reaction (RT-PCR), because it is expressed in lymphomagenesis with T cell lines and primary tumoral T cells.

The current report calls attention to the importance of the early diagnosis of MEITL that will enable proper management, essential for radical treatment, and we also describe some of the features that help distinguish MEITL from other intestinal T-cell lymphomas.

## 2. Case report

### 2.1. Clinical findings

We report a case of a 70-years-old male patient who was admitted to “St. Apostol Andrei” Emergency County Hospital in Constanta, Romania, with abdominal pain in the right upper part of the abdomen, transit disorder and weight loss (10 kg/6 months). The local examination revealed a palpable mass localized in the right abdominal flank.

His routine blood investigations showed a secondary anemia (9,4 g/dL hemoglobin, hematocrit 32,3%, and mean corpuscular volume 84,8 fl), with thrombocytopenia grade 3 (platelet 48,000/μL) and hypoalbuminemia (2,3 g/dL). The white blood cells, LDH, CRP, and alkaline phosphatase levels, blood chemistry, carcinoembryonic antigen, carbohydrate antigen 19-9, and alpha-fetoprotein tumor markers were all within normal limits. Upper GI endoscopic examination revealed gastroduodenitis and a gastric ulcer. Endoscopic examination of the colon revealed a polyp of the ascending colon that was removed and sent to the pathology department. The histological investigation of the colonic polyp revealed a tubular adenoma with low-grade dysplasia.

**Figure 1.**
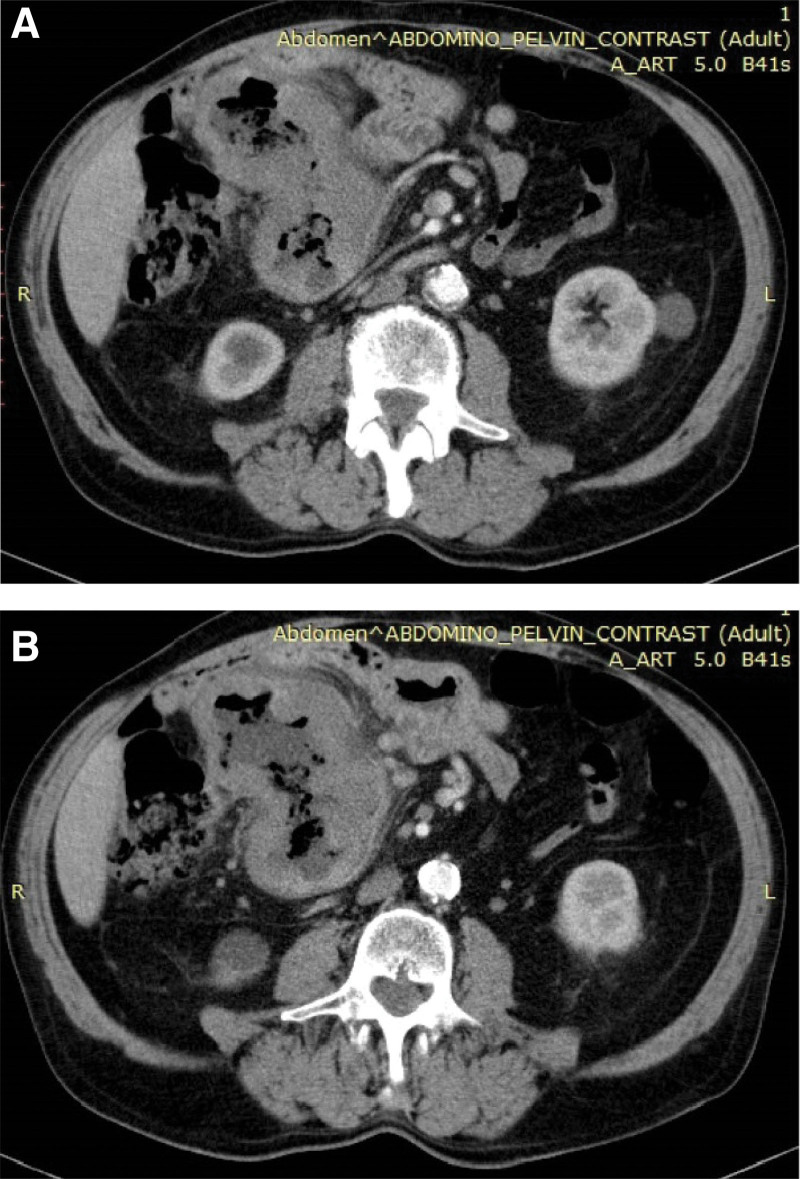
Abdominal computed tomography (CT) scan at the first diagnosis, with intravenous administration of contrast material. (A) The image demonstrates an ileal loop with thickened, iodophilic, irregular walls, located adjacent to the ascending colon, with infiltration of adjacent adipose tissue. (B) The image shows adenopathies and mesenteric adenopathic blocks (maximum 3.9 × 2.6 cm).

Computed tomography (CT) of the abdomen and pelvis at the initial diagnosis, with intravenous administration of contrast material showed an ileal loop with thickened, iodophilic, irregular walls, located adjacent to the ascending colon, with infiltration of adjacent adipose tissue, another ileal loop with the same appearance in the right iliac fossa, adenopathies and mesenteric adenopathic blocks (maximum 3.9 × 2.6 cm) and minimum pneumoperitoneum in the right flank and minimal ascites in the pouch of Douglas (Fig. 1).

Following the clinical and imagistic investigations, the patient subsequently underwent small bowel and large bowel resection with ileotransverse anastomosis and latero-lateral enteroanastomosis. The surgical specimens were sent to the Clinical Service of Pathology for morphological evaluation.

He was treated with 6 cycles of (appropriate) chemotherapy with cyclophosphamide, doxorubicin, etoposide, vincristine, and prednisone. Follow-up abdominal CT evaluation after 3 months showed no disease progression. CT evaluation of the chest showed residual pulmonary nodules and an adenoma of the left adrenal gland. Multiple polymerase chain reaction (PCR) tests for COVID-19 were performed, with negative test results.

The patient was discharged from the hospital in a good medical condition.

### 2.2. Histopathological examination

**Figure 2.**
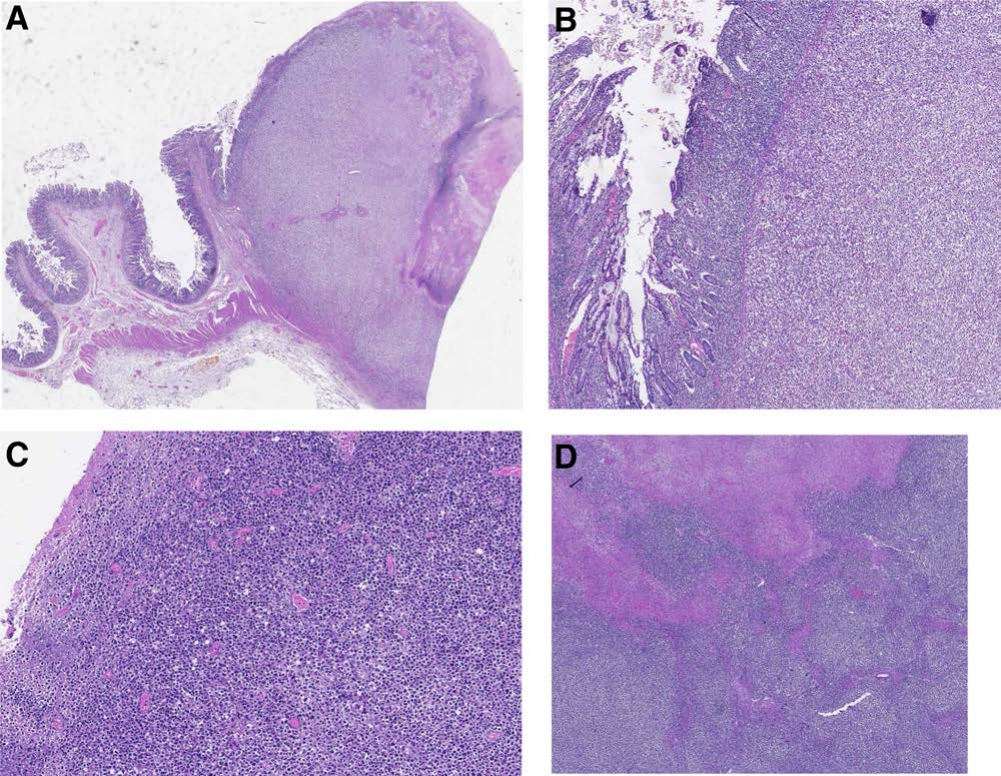
Monomorphic epitheliotropic intestinal T-cell lymphoma with metastasis in the mesenteric lymph node (H&E, 40X): (A, B, C): The images demonstrates a malignant intestinal lymphoid proliferation, with medium and large size of cells, relatively monomorphic, with vesicular nuclei, with prominent nucleoli and weak eosinophilic/clear cytoplasm. (D) The image shows a mesenteric lymph node with blurred architecture by the presence of a neoplastic cellular infiltrate with the same features described at the enteral level and with important areas of necrosis.

Macroscopically, 3 ulcero-vegetant and infiltrative lesions of 48.5, 2.5 × 4, and 8 × 11 cm were seen arising from the enteral mucosa. The selected surgical specimens were fixed in 10% formalin and paraffin-embedded, then stained with hematoxylin eosin. Microscopic examination (Fig. 2) showed the small intestinal lesions to consist of malignant lymphoid proliferation, with intermediate- to large- sized cells, relatively monomorphic, with vesicular nuclei, with prominent nucleoli and weak eosinophilic/clear cytoplasm. The presence of Reed-Sternberg-type uni-/binucleate cells was sporadically associated.

The described cell population was surrounded by a polymorphic inflammatory background (lymphocytes, plasma cells, frequent eosinophilic granulocytes, macrophages) with a diffusely transmural disposition, with formation of lympho-epithelial lesions, mucosal ulceration, and extension to the adjacent colic loops.

Key areas of necrosis, vascular thrombosis, and endothelitis were identified.

The mesenteric lymph nodes showed partial involvement by the same atypical lymphoid cells noted in the small intestine. Ileal and colonic resection margins were free, and it was also found hypertrophy and hyperplasia of the lymphoid follicles of the appendix.

The slides were evaluated by a Nikon Eclipse E600 microscope and representative photos were taken from digital whole slide images, obtained with HuronTISSUEScope^TM^ 4000XT scanner.^[11]^

### 2.3. Immunohistochemistry evaluation

**Figure 3.**
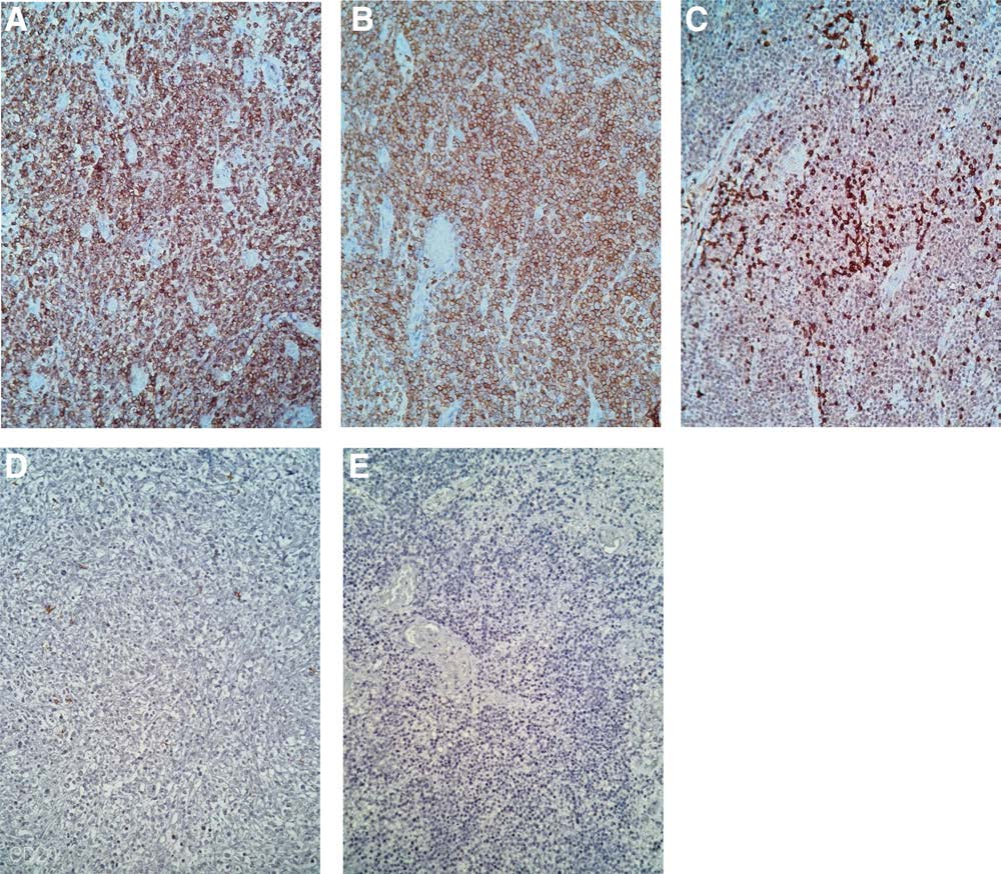
Immunohistochemistry evaluation of the surgical specimen from the enteral lesions. (A) The image shows 95% positive membranous and cytoplasmic immunonostain for CD3 biomarker (IHC; 40X). (B) The image demonstrates 95 % positive cytoplasmic immunostain for CD8 (IHC; 40X). (C) CD5 biomarker positive in scattered cells within the tumor (IHC; 40X). (D) Negative immunostain for CD20 biomarker (IHC; 40X). (E) The image shows focally positive cells for CD30 biomarker (IHC; 40X).

Further to the histopathological examination, immunohistochemical testing was mandatory, to establish the proliferation line. Immunohistochemical evaluation (Fig. 3) was performed on 4-μm thick sections of a representative formalin-fixed, paraffin embedded tissue block from the enteral lesion samples. After epitope retrieval, tissue sections were incubated with a panel of 5 monoclonal mouse antibodies, ready to use from BIOCARE Medical (Table 1).

**Table 1:** Antibodies used for immunohistochemical evaluation.

Antibody	Isotype	Clone	Antigen retrieval	Dilution	External control
CD3	IgG2a	PS1	CD3 (T-cell)	1:50	Tonsil or T-cell lymphoma
CD5	IgG1/kappa	4C7	CD5	1:100	Mantle cell lymphoma
CD8	IgG1/kappa	C8/144B	CD8	1:50	Tonsil or normal colon
CD20	IgG2a/kappa	L26	CD20 (B-cell)	1:100	Tonsil or B-cell lymphoma
CD30	IgG2a	CON6D/B5	CD30	1:100	Hodgkin’s or anaplastic large cell lymphoma

CD2, CD3, CD5, CD8, CD20, CD30, CD56 = antibodies.

In the present study, immunophenotyping showed the intermediate- to large-sized cells to be of T cell origin with strongly CD3 (Fig. 3A) and CD8 (Fig. 3B) positive; CD5 was positive in scattered cells within the tumor (Fig. 2C) and focally positive cells for CD30 biomarker (Fig. 3E) were observed. Negative results were noticed for CD20 biomarker (Fig. 3D). Based on the morphology and immunoprofile, a diagnosis of monomorphic epitheliotropic intestinal T-cell lymphoma with Reed-Sternberg-like cells was made.

### 2.4. Genomics

Pim oncogenes, which do have hematological and epithelial origins, are overexpressed in a variety of tumors. Serine/threonine kinases that are encoded by Pim genes have been proved to be able to inhibit the enhanced sensitivity to apoptosis induction that is connected to MYC-driven carcinogenesis. Characterizing the PIM-mediated survival signaling pathways has advanced significantly recently. These oncogenes may be attractive targets for highly specific and selective treatments with favorable toxicity profiles due to the distinctive structure of their active sites and the limited phenotype of animal mutants for all Pim family members.

Serine/threonine kinases with constitutive activity are known as Pim kinases (and therefore lack the need for posttranslational activation). The activity of Pim kinases is mostly controlled at the transcriptional and translational stages since their mRNA and proteins have a truly short half-life.

### 2.5. Quantitative RT-PCR, ribonucleic acid extraction and results of quantification

Total ribonucleic acid was extracted and purified using the extraction kit RNeasy formalin fixed paraffin embedded Tissue Kit (Qiagen, Germany) in accordance with the manufacturer’s instructions to measure the ribonucleic acid levels of this gene in MEITL cell lines, primary tumoral, and normal T cells as well as to assess the efficacy of the PIM1 gene ‘knockdown and evaluate the levels of this gene in MEITL cell lines. Quantitative RT-PCR was used to gauge the PIM1 gene’s expression. The complementary DNA that resulted was put in a plate with a Taqman probe (TaqMan SNP Assay for PIM1). Under the following thermal cycler settings, PCR amplification was conducted using the Applied Biosystems - Sistem Fast Real Time PCR 7500 Sequence Detection System (Life Technologies). Relative quantification was computed using the CT technique, and the outcome showed that the PIM1 gene has low expression.

## 3. Discussions

This current case report stood for a primary T-cell lymphoma with immunophenotype features consistent with monomorphic epitheliotropic T-cell lymphoma.

Recent data have led to changes in the categorization of intestinal T-cell lymphomas, and it has become apparent that the 2 subtypes formerly designated as variants of EATL are distinct.^[1]^ Type I EATL, now simply designated as EATL, is closely linked to celiac disease and is primarily a disease of individuals of northern European origin. Type II EATL, now formally designated as monomorphic epitheliotropic intestinal T-cell lymphoma, shows no association with celiac disease and appears relatively increased in incidence in Asian and Hispanic populations.^[1]^ MEITL generally is positive for CD3, CD8, and negative for CD5 and CD30.^[1]^

There are several reports with conflicting opinions about Type II EATL and according to a study from the International T-cell Lymphoma Project, both types of EATL are associated with celiac disease, although multiple reports from Asia showed no such association.^[3,4,12–18]^ Although there is no association between MEITL and celiac disease, recent reports have suggested some cases of MEITL to be preceded by a variant of celiac disease.^[19]^ Moreover, in this present case of T-cell lymphoma, there is no association between celiac disease and MEITL.

About the phenotype of this lymphoma, MEITL was originally described as an intestinal T-cell lymphoma with a CD8 + CD56 + phenotype, but according to multiple studies neither marker is mandatory for diagnosis and cases have been reported lacking either or both these molecules.^[4,12,13,16,20–23]^

Lymphocytes in the intra-epithelial compartment are described as having a phenotype that is similar to or consistently discordant with that of the invasive tumor^[15,22]^ and they must also express some pan-T-cell markers such as CD2, CD3, CD5, CD7. Expression of CD8 and CD56, although typical for this neoplasm, was not considered mandatory for the diagnosis.^[12,13,16,21]^

In this current case report, immunophenotyping showed a positive expression of CD3, CD5, CD8 and confirms earlier studies about typical expression of CD8 and other T-cell markers for the diagnosis of MEITL.

According to Delabie et al, the small intestine was the most commonly involved site (90% of cases) in EATL and MEITL patients and high serum LDH and CRP levels may reflect a high tumor burden and extensive tissue damage, which may explain the adverse prognosis.^[3]^ The above-mentioned study proved that 65% (37/57) of MEITL/EATL cases did not have an elevated LDH level.^[3]^ In the current case, we noticed that the patientʼs laboratory investigations showed a normal CRP and LDH at admission to our hospital, in spite of widespread lesions. Consequently, we should consider the possibility of malignant lymphoma including MEITL and the elevated risk of intestinal perforation, even in cases with low CRP levels and LDH, because an early diagnosis of MEITL may contribute to the prevention of perforation and implementation of successful chemotherapy.^[24]^

Inflammatory bowel disease, indolent T-cell lymphoproliferative disease, EATL, and intestinal natural killer/T-cell lymphoma can be the differentials of MEITL and the most important features that can guide us to differentiate MEITL from other types of T-cell lymphoma are the epitheliotropic patterns, the monomorphic cell shapes and positive immunophenotyping for CD8.^[2,25]^

## 4. Conclusions

In conclusion, we described a rare case of MEITL, with low expression of gene PIM1, that was correctly diagnosed based on the typical immunophenotype features, epitheliotropic patterns and monomorphic cell shape and in view of the differences in epidemiology and clinicopathologic features, we strongly believe that it is justified to separate out type II EATL from the EATL category as a distinct form of lymphoma, with the lack of association with celiac disease.

## Acknowledgments

This research was performed in the Center for Research and Development of the Morphological and Genetic Studies of Malignant Pathology from the “Ovidius” University of Constanţa.

## Author contributions

All authors critically revised the manuscript, approved the final version to be published, and agree to be accountable for all aspects of the work.

**Conceptualization:** Mădălina Boșoteanu, Miruna Cristian, Anca Florentina Mitroi, Costel Stelian Brînzan.

**Formal analysis:** Mădălina Boșoteanu, Miruna Cristian, Mariana Așchie, Gabriela Izabela Bălțătescu.

**Investigation:** Miruna Cristian, Anca Florentina Mitroi, Costel Stelian Brînzan.

**Methodology:** Miruna Cristian, Anca Florentina Mitroi, Costel Stelian Brînzan.

**Supervision:** Mădălina Boșoteanu, Mariana Așchie, Mariana Deacu, Gabriela Izabela Bălțătescu.

**Validation:** Mădălina Boșoteanu, Mariana Așchie, Mariana Deacu, Anca Florentina Mitroi, Costel Stelian Brînzan.

**Visualization:** Anca Florentina Mitroi.

**Writing – original draft:** Mădălina Boșoteanu, Miruna Cristian, Anca Florentina Mitroi, Costel Stelian Brînzan.

**Writing – review & editing:** Mădălina Boșoteanu, Miruna Cristian, Mariana Așchie, Mariana Deacu, Gabriela Izabela Bălțătescu.
